# Effect of Date Palm (*Phoenix dactylifera*) Phytochemicals on Aβ_1−40_ Amyloid Formation: An *in-silico* Analysis

**DOI:** 10.3389/fnins.2022.915122

**Published:** 2022-07-25

**Authors:** Qamar Zia, Md Tabish Rehman, Md Amiruddin Hashmi, Sahabjada Siddiqui, Abdulaziz Bin Dukhyil, Mohammad Z. Ahmed, Azfar Jamal, Saeed Banawas, Sami G. Almalki, Mohammad Owais, Hamad Qasem Aldhafeeri, Ibrahim M. Ibrahim, Wael Alturaiki, Mohamed F. AlAjmi, Mohammed Alsieni, Yaser E. Alqurashi

**Affiliations:** ^1^Department of Medical Laboratory Sciences, College of Applied Medical Sciences, Majmaah University, Al Majmaah, Saudi Arabia; ^2^Health and Basic Sciences Research Center, Majmaah University, Al Majmaah, Saudi Arabia; ^3^Department of Pharmacognosy, College of Pharmacy, King Saud University, Riyadh, Saudi Arabia; ^4^Interdisciplinary Biotechnology Unit, Faculty of Life Sciences, Aligarh Muslim University, Aligarh, India; ^5^Department of Biotechnology, Era's Lucknow Medical College and Hospital, Era University, Lucknow, India; ^6^Department of Biology, College of Science Al-Zulfi, Majmaah University, Majmaah, Saudi Arabia; ^7^Department of Biomedical Sciences, Oregon State University, Corvallis, OR, United States; ^8^Department of Pharmacology, Faculty of Medicine, King Abdulaziz University, Jeddah, Saudi Arabia

**Keywords:** Alzheimer's disease, *Phoenix dactylifera* (date palm), molecular docking (MD), phytochemicals (alkaloids/lignans), drug likeness and bioactivity

## Abstract

Alzheimer's disease (AD) is a neurodegenerative disease and the most prevalent form of dementia. The generation of oxygen free radicals and oxidative damage is believed to be involved in the pathogenesis of AD. It has been suggested that date palm, a plant rich in phenolic compounds and flavonoids, can provide an alternative treatment to fight memory loss and cognitive dysfunction due to its potent antioxidant activity. Thus, we studied the effect of flavonoids present in date palm on Aβ_1−40_ amyloid formation using molecular docking and molecular dynamics simulation. AutoDock. Myricetin was used as a positive control drug. The flavonoids Diosmetin, Luteolin, and Rutin were found to be potent inhibitors of aggregation (docking energies ≤ −8.05 kcal mol^−1^) targeting Aβ_1−40_ fibrils (both 2LMO and 6TI5), simultaneously. Further screening by physicochemical properties and drug-likeness analysis suggested that all flavonoids except Rutin followed Lipinski's rule of five. Rutin was, thus, taken as a negative control (due to its violation of Lipinski's rule) to compare its dynamics with Diosmetin. Diosmetin exhibited the highest positive scores for drug likeness. Since Luteolin exhibited moderate drug-likeness and better absorption properties, it was also included in molecular dynamics simulation. Molecular dynamics of shortlisted compounds (Rutin, Diosmetin, and Luteolin) were performed for 200 ns, and the results were analyzed by monitoring root mean square deviations (RMSD), root mean square fluctuation (RMSF) analysis, the radius of gyration (Rg), and solvent accessible surface area (SASA). The results proved the formation of a stable protein-compound complex. Based on binding energies and non-bonded interactions, Rutin and Luteolin emerged as better lead molecules than Diosmetin. However, high MW (610.5), lowest absorption rate (16.04%), and more than one violation of Lipinski's rule make Rutin a less likely candidate as an anti-amyloidogenic agent. Moreover, among non-violators of Lipinski's rule, Diosmetin exhibited a greater absorption rate than Luteolin as well as the highest positive scores for drug-likeness. Thus, we can conclude that Diosmetin and Luteolin may serve as a scaffold for the design of better inhibitors with higher affinities toward the target proteins. However, these results warrant *in-vitro* and *in-vivo* validation before practical use.

## Introduction

Alzheimer's disease (AD), a progressive neurodegenerative disorder, is most prevalent among the elderly and encompasses cognitive dysfunction, intellectual decline, and personality changes (Yamada et al., 1999). AD is typically associated with granulovacuolar degeneration, amyloid precursor protein (APP) derived amyloid-beta (Aβ) peptide deposition in extracellular tissue, and neurofibrillary tangles (NFTs) within the neurons. AD and other forms of dementia are ranked as the 7th leading cause of death globally according to the World Health Organization (WHO) (WHO Fact Sheet, 2020), with 40–50 million individuals currently living with dementia (Nichols et al., 2019). The prevalence of dementia in Saudi Arabia is estimated at 6.4%, with number of cases projected to nearly triple by 2060 [Ministry of Health (MoH), KSA, 2022]. As the elderly population ages, Alzheimer's, a form of dementia, is likely to become a more significant healthcare issue, if proactive measures are not taken (Alzheimer's Association Report, 2020).

Although the pathogenesis of AD is complex, increased oxidative distress forms the basis for neurodegeneration (Markesbery and Carney, 1999). Memory-related brain structures are particularly susceptible to oxidative stress because they require a high amount of oxygen (Floyd, 1992; Coyle and Puttfarcken, 1993). Highly reactive oxygen free radicals generated during high metabolic activity in the brain are toxic to neuronal cells; thus, believed to be involved in the etiology of the disease. Aging increases chronic oxidative stress, a major risk factor for Alzheimer's (Lee et al., 2022).

Several epidemiological studies investigating the effect of dietary components on AD are in the early stages. Nonetheless, fruit- and vegetable-rich diet may provide an effective alternative to AD by improving age-related memory decline and cognitive dysfunction associated with AD (Dominguez and Barbagallo, 2018). In the animal model, the antioxidant nutrients appear to protect neurons from oxidative damage and inflammatory responses. Histological studies also indicated that mice fed with antioxidant supplements exhibit less neuronal cell death (Joseph et al., 1998; Guerrero et al., 1999).

Fruits of the date palm (*Phoenix dactylifera* L. Arecaceae) represent a vital component of the diet and a staple food in Arabian countries. Date fruit is listed in folk remedies for the treatment of various diseases (Duke, 1992). In addition, date palm fruits have demonstrated immunomodulatory (Puri et al., 2000), antibacterial (Sallal and Ashkenani, 1989), antihyperlipidemic (Salah and Al-Maiman, 2005), hepatoprotective (Saafi et al., 2011), renal protective (Al Qarawi et al., 2008), anticancerous (Ishurda and John, 2005), antifungal (Sallal et al., 1996; Shraideh et al., 1998), and antimutagenic activities (Vayalil, 2002). The importance of dates in human nutrition derives from its valuable ingredients, such as carbohydrates, dietary fiber, salts, vitamins, and proteins (Vayalil, 2002). Besides nutritional value, date fruits are rich in antioxidants and phenolic compounds with free radical activity. An aqueous extract of date palm (ADFE) has recently shown promising neuroprotective activity in different models of neurodegeneration (Asadi-Shekaari et al., 2008; Zangiabadi et al., 2011; Badeli et al., 2016).

This study was designed to investigate the anti-amyloidogenic property of flavonoids present in date palm extract. We tested several date palm compounds against the Aβ_1−40_ fibrils, responsible for the formation of amyloid. We also evaluated drug-likeness and toxicity potential of these chemicals Molecular docking was then performed to ascertain the best ligand. Next, we assessed its binding potential and stability in molecular dynamics studies. This study suggests that Diosmetin may be used as a novel inhibitor of protein aggregation and can act as a neuroprotective agent.

## Materials and Methods

### Preparation of Proteins and Ligands

The protein targets used in this study [2LMO: structural model of a 40-residue β-amyloid fibril, and 6TI5: structural model of Aβ_1−40_ fibrils] were downloaded from the PDB RCSB database (www.rcsb.org). It was noted that there are no water molecules and heteroatoms in the pdb files of 2LMO and 6TI5. Thus, prior to molecular docking, the proteins were pre-processed only by assigning Kollman charges using AutoDock Tool (ADT). The structure of protein molecules was finally energy minimized by MMFF (Merck Molecular Force Field) using Discovery Studio. The 2D structures of ligands namely, Apigenin (CID: 5280443), Cianidanol (CID: 9064), Diadzein (CID: 5281708), Diosmetin (CID:5281612), Ferulic acid (CID: 445858), Formonometin (CID:5280378), Gallic acid (CID:370), Genistein (CID:5280961), Gycitein (CID:5317750), Luteolin (CID:5280445), Quercetin (CID:5280343), Rutin (CID:5280805), Sinapic acid (CID:637775), and Vanillic acid (CID:8468) were downloaded from PubChem database and prepared for molecular docking by assigning bond orders and angles using ADT. Gasteiger partial charges were defined in ADT, and the energies of all the ligands were minimized using UFF (Universal Force Field).

### Molecular Docking

The interaction between proteins and ligands was determined by conducting molecular docking using AutoDock4.2 (Morris et al., 2009; Alsaleem et al., 2020; Al-Shabib et al., 2020). All the ligands were individually docked with each of the target proteins in separate docking runs. The molecular docking was performed inside a grid box covering the whole protein molecule i.e., a blind docking approach was adopted. For 2LMO, the dimension of the grid box was set to 50.7 × 77.7 × 58.9 Å, centered at 13.8 × 69.8 × 72.3 Å with 0.375 Å spacing between the grid points. Similarly, the dimension of the grid box for 6TI5 was set to 45.4 × 52.1 × 58.6 Å, centered at −1.1 × 6.0 × 1.6 Å with 0.375 Å spacing. Molecular docking was performed using LGA (Lamarck Genetic Algorithm) and Solis-Wets local search methods During molecular docking, LGA is generally used as the global search method, while the Solis-Wets method is directed for the local search. Solis and Wets local search act as a kind of cross-validation of the free energy model (Morris et al., 1998). For each run, 2.5 × 10^6^ energy calculations were computed and a total of 10 docking runs were performed. The population size, translational step, quaternions, and torsions were set as 150, 0.2, 5, and 5 respectively. The van der Waals' and electrostatic parameters were calculated with the help of a distance-dependent dielectric function. We have also performed molecular docking using DockThor using the default setting to reconfirm the results of AutoDock4.2 (Guedes et al., 2021). The docking affinity or dissociation constant (*K*_d_) of ligands for proteins was estimated from docking energy (Δ*G*) using the following relation as reported earlier (Ahmed et al., 2021).


(1) $$\begin{array}{r} \Delta G=-RT\ln\;K_{d} \end{array}$$

where, *R* and *T* were universal gas constant (1.987 cal/mol-K) and temperature (298 K) respectively.

### Calculation of Physicochemical Properties and Prediction of Toxicity Potential

Drug-likeness, mutagenic, tumorigenic, reproductive, and irritant effects of drug-toxicity risk parameters were analyzed by OSIRIS Data Warrior V5.2.1 software (https://openmolecules.org/datawarrior/) (Siddiqui et al., 2020; Iqbal et al., 2022). All of the 14 active constituents of date palm were also evaluated using Lipinski's rule of five (Lipinski, 2004). The drug-likeness parameters *viz*. MW ≤ 500, logP ≤ 5, number of hydrogen bond donors (NOHNH) ≤ 5 and hydrogen bond acceptor sites (NON) ≤ 10, topological polar surface area (TPSA) (≤ 140 Å^2^), and number of the rotatable bond (≤ 10) were measured. The absorption % was calculated as: % Absorption = 109 – [0.345 × Topological Polar Surface Area] (Zhao et al., 2002).

### Molecular Dynamics Simulation

The molecular dynamics (MD) simulation of the 2LMO and 6TI5 protein and their respective complexes with Diosmetin, Luteolin, and Rutin exhibiting the lowest binding energies were performed in the aqueous environment. The MD simulations were carried out in Gromacs-2018.1 using the Amber99SB-ILDN force field (Van Der Spoel et al., 2005). All the 3 ligand molecules were extracted from their respective complexes with 2LMO and 6TI5 and their topologies were generated in the AmberTools21 using the AM1-BCC charge model with Antechamber packages (Sousa Da Silva and Vranken, 2012). Both the 2LMO and 6TI5 protein alone and their complexes with Diosmetin, Luteolin, and Rutin were first solvated using the TIP3P water model followed by the neutralization of charges of each system by adding an equal number of counter sodium/chlorine ions. All systems were minimized to a maximum of 50,000 steps using the steepest descent minimization to remove the weak Van der Waals contacts. The first equilibration of all systems (NVT equilibration) was done using a V-rescale thermostat at 300 K and constant volume for 200 ps at a coupling constant of 0.1 ps (Bussi et al., 2007). The second equilibration (NPT equilibration) was performed using Parrinello–Rahman barostat at 1.0 bar and 300 K for 200 ps having a coupling constant of 2 ps (Parrinello and Rahman, 1981). The Coulombs and Lennard Johns interaction had a cutoff distance of 1.4 nm with an integration time step of 2 fs (Darden et al., 1993). The electrostatic interaction was governed using PME (Particle Mesh Ewald) and the Fourier transformation had a grid spacing of 0.16 nm (Essmann et al., 1995). Finally, 200 ns production MD simulation of a total of eight systems, including 2LMO and 6TI5 protein alone and the respective complexes for each protein with Diosmetin, Luteolin, and Rutin, was performed in which 20,000 frames of each trajectory were recorded. The trajectories were subjected to PBC corrections before the analysis. The MM-PBSA analysis for the interaction of the three ligand molecules with the 2LMO and 6TI5 protein was performed for the evaluation of the binding energies (Kumari et al., 2014).

## Results and Discussion

In AD, isoforms of different lengths of β-amyloid protein (Aβ) derived from endoproteolytic cleavage of the transmembrane APP, are the main components of senile plaques (Henning-Knechtel et al., 2020). Aβ monomers aggregate into different forms of oligomers, which can then form fibrillar polypeptide aggregates found in the brains of Alzheimer's disease patients (Chen et al., 2017). The 40-residue peptide Aβ_1−40_ represents the most abundant Aβ isoform in the brain (Mori et al., 1992; Selkoe and Hardy, 2016). It has been established that high Aβ_1−40_ levels are associated with a greater mortality rate in the elderly (Lehmann et al., 2020). Therefore, we have selected Aβ_1−40_ as our model protein. Since diverse conformations of the same protein are available, we choose to perform our studies on two different conformers of the same target (Aβ_1−40_) namely, 2LMO and 6TI5.

Dates are a good source of energy, vitamins, and important elements, such as phosphorus, iron, potassium, and a significant amount of calcium (Aljaloud et al., 2020). Dates have been reported to have high antioxidant contents and activities (Saleh et al., 2011; Mistrello et al., 2014; Al-Jasass et al., 2015; Shahdadi et al., 2015). Studies with various varieties of dates have shown the presence of both free and bound phenolic acids (Al-Farsi et al., 2005) that are responsible for their potent antioxidant property. Moreover, date varieties from different regions had different levels and patterns of phenolic acids. Various phenolic acids (Luteolin, quercetin, Rutin, apigenin, (+)-catechin, and (–)-epicatechin, gallic, p-hydroxybenzoic, vanillic, caffeic, syringic, sinapic, coumaric, ferulic and protocatechuic acid) have been tentatively identified (Al-Shwyeh, 2019). Here, we speculated whether date palm fruits growing in Saudi Arabia can inhibit the formation of the Aβ1-40 fibril. For this, we evaluated the drug-likeness and toxicity potential of common date palm phytocomponents. The best ones were then subjected to molecular docking and simulation studies to identify the paramount compound that can be used against Aβ_1−40_ fibrils. We have included Myricetin as a control/standard ligand in molecular docking with both target proteins. Also, we have performed molecular docking using DockThor to confirm the results obtained using AutoDock.

### Docking of Natural Compounds of Date Palm Fruits Against 2LMO and 6TI5

In this study, the binding affinities of various natural compounds as promising anti-aggregation lead molecules against Aβ_1−40_ were determined by molecular docking. The computational screening revealed the AutoDock docking energies of the studied ligands were in the range of −6.2– −8.7 kcal/mol, and −5.5– −8.5 kcal/mol for 2LMO and 6TI5, respectively (Table 1). Moreover, the docking energies obtained from DockThor server were in the range of −6.3– −8.9 kcal/mol for 2LMO and −6.5– −8.3 kcal/mol for 6TI5 (Table 1). Based on the docking score of ligands from AutoDock, Diosmetin, Genistein, Gycitein, Luteolin, and Rutin had binding energies ≤ −8.5 kcal/mol (docking energy of Myricetin) against 2LMO. Likewise, Diosmetin, Genistein, Luteolin, and Rutin displayed docking energies ≤ −7.6 kcal/mol (docking energy of Myricetin) against 6TI5. Further analysis by comparing the docking energies of ligands obtained from DockThor revealed that three ligands (Diosmetin, Luteolin, and Rutin) showed binding energies of ≤ −8.5 kcal/mol (which is the docking energy of the control ligand i.e., Myricetin) against 2LMO. Similarly, Diadzein, Diosmetin, Formononetin, Genistein, Luteolin, Quercetin, and Rutin displayed binding energies of ≤ −7.6 kcal/mol (docking energy of Myricetin) against 6TI5. An analysis of these results showed that Diosmetin, Luteolin, and Rutin were the most promising natural compounds targeting both 2LMO and 6TI5, simultaneously. Hence, a detailed interaction and molecular dynamics simulation of Diosmetin, Luteolin, and Rutin was further studied.

**Table 1:** Molecular docking scores of selected palm date phytochemicals.

**S. No**.	**Compound name**	**PubChem ID**	**Formula**	**AutoDock docking energy (kcal mol** ^**−1**^ **)**		**DockThor docking energy (kcal mol** ^**−1**^ **)**	
				**2LMO**	**6TI5**	**2LMO**	**6TI5**
1.	Apigenin	5280443	C_15_H_10_O_5_	−8.0	−7.2	−8.4	−7.1
2.	Cianidanol	9064	C_15_H_14_O_6_	−8.3	−7.3	−8.2	−7.2
3.	Diadzein	5281708	C_15_H_10_O_4_	−7.6	−7.9	−8.3	−7.3
4.	Diosmetin	5281612	C_16_H_12_O_6_	−8.5	−7.7	−8.6	−7.8
5.	Ferulic acid	445858	C_10_H_10_O_4_	−6.3	−6.2	−6.8	−6.7
6.	Formononetin	5280378	C_16_H_12_O_4_	−7.9	−8.5	−8.4	−7.2
7.	Gallic acid	370	C_7_H_6_O_5_	−6.2	−5.6	−6.3	−6.6
8.	Genistein	5280961	C_15_H_10_O_5_	−7.6	−7.8	−8.9	−7.7
9	Gycitein	5317750	C_16_H_12_O_5_	−7.7	−7.1	−8.5	−7.2
10.	Luteolin	5280445	C_15_H_10_O_6_	−8.5	−7.7	−8.7	−7.9
11.	Quercetin	5280343	C_15_H_10_O_7_	−8.2	−8.0	−7.1	−6.9
12.	Rutin	5280805	C_27_H_30_O_16_	−8.7	−8.5	−8.9	−8.3
13.	Sinapic acid	637775	C_11_H_12_O_5_	−6.3	−5.5	−6.9	−6.8
14.	Vanillic acid	8468	C_8_H_8_O_4_	−6.5	−5.6	−6.8	−6.5
15.	Myricetin (Control)	5281672	C_15_H_10_O_8_	−8.5	−7.6	−8.5	−7.6

### Prediction of Physicochemical Properties, Drug-Likeness, and Toxicity Potentials

Analysis of toxicity risk assessment provides the initial knowledge of probable side effects of phytocomponents that may be utilized in lead discovery and development. The prediction of different properties of phytocomponents at an early stage is a vital step in leading discovery and development. The OSIRIS Data Warrior V5.2.1 program was used to assess the toxicological characteristics and drug-likeness of date palm phytochemicals. Lipinski's rule explains the molecular characteristics of a chemical that are critical for lead optimization and selectivity of a possible orally active therapeutic candidate in clinical applications (Lipinski, 2004). In general, an orally active drug should have no more than one Lipinski violation, otherwise, its bioavailability will be reduced. Among all compounds, Rutin displayed three violations of Lipinski's rule of five (Table 2). The lowest absorption rate (16.04%) was expected for Rutin due to its high MW (610.5), making it a less likely candidate as an anti-amyloidogenic agent. Moreover, all of the compounds having a molecular mass of <500 g/mol, showed high gastrointestinal absorption and zero violation of Lipinski's rule. Among non-violators of Lipinski's rule, Quercetin exhibited the lowest absorption rate. Considering the analyzed physicochemical properties and absorption potential, a further toxicological investigation was carried out and found that Formononetin, Sinapic acid, Cianidanol, Diosmetin, Rutin, and Luteolin exhibited no toxicity for all the tested parameters (Table 3). However, the dug-likeness was very low for Formononetin. Diosmetin exhibited the highest positive scores for drug likeness. This stimulated us to explore its property to ameliorate AD and was, therefore, selected for molecular docking and molecular dynamics simulation analysis. We also studied Rutin as a negative control (due to its violation of Lipinski's rule) to compare its dynamics with Diosmetin. Since Luteolin exhibited moderate drug-likeness and better absorption properties, it was also included in molecular dynamics simulation.

**Table 2:** Physicochemical properties of palm date phytochemicals.

**S. No**.	**Compound name**	**% Absorption (> 50%)**	**TPSA ()(<160)**	**MW** **(<500)**	**c logP (<5)**	**HA**	**HBD (≤5)**	**HBA** **(≤10)**	**RB (≤10)**	**ROF violation**
1.	Apigenin	77.64	90.89	270.2	2.46	20	3	5	1	0
2.	Cianidanol	70.92	110.4	290.3	1.37	21	5	6	1	0
3.	Diadzein	84.61	70.67	254.2	2.56	19	2	4	1	0
4.	Diosmetin	74.45	100.1	300.3	2.28	22	3	6	2	0
5.	Ferulic acid	85.96	66.76	194.2	1.25	14	2	4	3	0
6.	Formononetin	88.41	59.67	268.3	3.1	20	1	4	2	0
7.	Gallic acid	75.19	97.98	170.1	0.59	12	4	5	1	0
8.	Genistein	77.64	90.89	270.2	2.27	20	3	5	1	0
9.	Glycitein	81.43	79.9	284.3	2.38	21	2	5	2	0
10.	Luteolin	70.66	111.1	286.2	1.97	21	4	6	1	0
11.	Quercetin	63.68	131.4	302.2	1.68	22	5	7	1	0
12.	Rutin	16.04	269.4	610.5	−1.06	43	10	16	6	3
13.	Sinapic acid	82.78	76	224.2	1.26	16	2	5	4	0
14.	Vanillic acid	85.96	66.76	168.2	1.19	12	2	4	2	0

*Percentage Absorption was calculated as: % Absorption = 109 – [0.345 × Topological Polar Surface Area]; TPSA, MW, HA, HBD, HBA, RB and ROF stands for total polar surface area, molecular weight, number of heavy atoms, number of hydrogen bond donors, number of hydrogen bond acceptors, number of rotatable bonds, and Lipinski's rule of five*.

**Table 3:** Drug-likeness and toxicity potential of palm date phytochemicals.

**S. No**.	**Compound name**	**Druglikeness properties**				
		**Druglikeness**	**Mutant**	**Tumorigenic**	**Reproductive effective**	**Irritant**
1.	Apigenin	0.28194	High	None	None	None
2.	Cianidanol	0.31525	None	None	None	None
3.	Diadzein	−0.09385	None	None	High	None
4.	Diosmetin	0.40331	None	None	None	None
5.	Ferulic acid	0.27506	High	High	High	None
6.	Formononetin	0.036465	None	None	None	None
7.	Gallic acid	−1.8442	High	None	High	None
8.	Genistein	−0.09385	High	High	High	None
9.	Glycitein	0.036465	None	None	High	None
10.	Luteolin	0.28194	None	None	None	None
11.	Quercetin	−0.08283	High	High	None	None
12.	Rutin	1.9337	None	None	None	None
13.	Sinapic acid	0.27506	None	None	None	None
14.	Vanillic acid	−1.597	High	None	None	None

### Molecular Docking Analysis

#### Interaction of 2LMO With Phytochemicals

**Figure 1:**
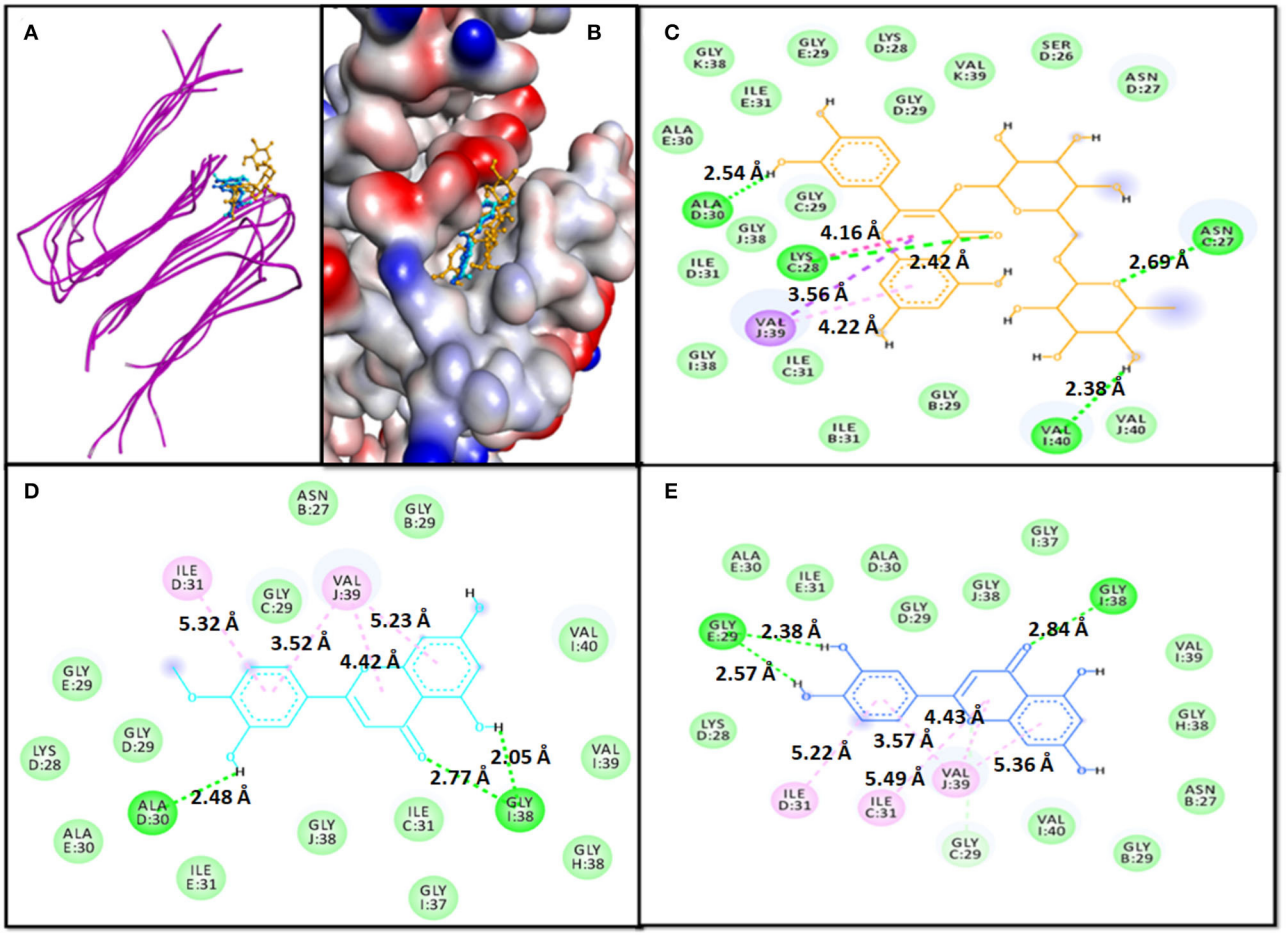
Molecular docking of 2LMO with phytochemicals. **(A)** 2D representation of the binding of phytochemical to 2LMO, **(B)** 3D representation of the binding of phytochemical to 2LMO, **(C)** Interaction between 2LMO and Rutin, **(D)** Interaction between 2LMO and Diosmetin, and **(E)** Interaction between 2LMO and Luteolin.

An analysis of molecular docking showed that Rutin, Diosmetin, and Luteolin were bound to a cavity created between different multiple chains of aggregated Aβ_1−40_ protein i.e. the 2LMO model (Figures 1A,B). The 2LMO–Rutin complex was primarily stabilized by hydrogen bonding and hydrophobic interactions. Rutin formed four hydrogen bonds with C:ASN27:HD22 (2.69 Å), C:LYS28:HN (2.42 Å), D:ALA30:O (2.54 Å), and I:VAL40:OXT (2.38 Å). Also, Rutin interacted with J:VAL39:CG2 (3.56 Å), C:LYS28:C, O;GLY29:N (4.16Å), and J:VAL39 (4.22 Å) through four hydrophobic interactions (Figure 1C). Some residues, such as D:ASN27, D:SER26, K:VAL39, D:GLY29, D:LYS28, E:GLY:29, E:ILE31, K:GLY38, E:ALA30, C:GLY29, J:GLY38, D:ILE31, I:GLY38, C:ILE31, B:ILE31, B:GLY29, and J:VAL40, are further stabilized 2LMO-Rutin complex by van der Waals' interactions. The binding free energy and the corresponding dissociation constant of 2LMO-Rutin were −8.7 kcal mol^−1^, and 2.40 × 10^6^ M^−1^ (Table 4).

**Table 4:** Parameters for the interaction of target proteins (2LMO and 6TI5) with Rutin, Diosmetin, and Luteolin as determined by molecular docking.

**Interaction between donor and acceptor atoms**	**Distance (Å)**	**Nature of interaction**	**Binding energy (Δ*G*), kcal mol^**−1**^**	**Binding affinity (*K*_d_), M^−1^**
***2LMO-Rutin***				
C:ASN27:HD22 - LIG:O C:LYS28:HN - LIG:O LIG:H - D:ALA30:O LIG:H - I:VAL40:OXT J:VAL39:CG2 - LIG C:LYS28:C,O;GLY29:N - LIG LIG - J:VAL39	2.69 2.42 2.54 2.38 3.56 4.16 4.22	Hydrogen Bond Hydrogen Bond Hydrogen Bond Hydrogen Bond Hydrophobic (Pi-Sigma) Hydrophobic (Amide-Pi Stacked) Hydrophobic (Pi-Alkyl)	−8.7	2.40 × 10^6^
***2LMO-Diosmetin***				
I:GLY38:HN - LIG:O LIG:H - D:ALA30:O LIG:H - I:GLY38:O LIG - J:VAL39 LIG - J:VAL39 LIG - D:ILE31 LIG - J:VAL39	2.77 2.48 2.05 3.52 4.42 5.32 5.23	Hydrogen Bond Hydrogen Bond Hydrogen Bond Hydrophobic (Pi-Alkyl) Hydrophobic (Pi-Alkyl) Hydrophobic (Pi-Alkyl) Hydrophobic (Pi-Alkyl)	−8.5	1.72 × 10^6^
***2LMO-Luteolin***				
I:GLY38:HN - LIG:O LIG:H - E:GLY29:O LIG:H - E:GLY29:O C:GLY29:CA - LIG:O LIG - C:ILE31 LIG - J:VAL39 LIG - J:VAL39 LIG - D:ILE31 LIG - J:VAL39	2.84 2.57 2.38 3.36 5.49 3.57 4.43 5.22 5.36	Hydrogen Bond Hydrogen Bond Hydrogen Bond Hydrogen Bond Hydrophobic (Pi-Alkyl) Hydrophobic (Pi-Alkyl) Hydrophobic (Pi-Alkyl) Hydrophobic (Pi-Alkyl) Hydrophobic (Pi-Alkyl)	−8.5	1.72 × 10^6^
***6TI5-Rutin***				
M:HIS13:ND1 - LIG:O LIG:H - F:ALA30:O LIG:H - K:VAL12:O F:LYS28:CE - LIG:O LIG:C - K:GLU11:OE2 LIG - E:ILE31	2.95 2.94 2.07 3.20 3.48 4.93	Hydrogen Bond Hydrogen Bond Hydrogen Bond Carbon Hydrogen Bond Carbon Hydrogen Bond Hydrophobic (Pi-Alkyl)	−8.5	1.72 × 10^6^
***6TI5-Diosmetin***				
LIG:H - M:GLU11:O K:GLU11:OE2 - LIG K:GLU11:OE2 - LIG L:GLU11:OE2 – LIG	2.38 3.98 3.38 4.22	Hydrogen Bond Electrostatic (Pi-Anion) Electrostatic (Pi-Anion) Electrostatic (Pi-Anion)	−7.7	4.44 × 10^5^
***6TI5-Luteolin***				
LIG:H - L:VAL12:O LIG:H - M:GLU11:O LIG:H - J:VAL12:O LIG:H - K:GLU11:O K:GLU11:OE2 - LIG K:GLU11:OE2 - LIG L:GLU11:OE2 – LIG	2.44 2.35 2.49 2.11 4.05 3.35 4.32	Hydrogen Bond Hydrogen Bond Hydrogen Bond Hydrogen Bond Electrostatic (Pi-Anion) Electrostatic (Pi-Anion) Electrostatic (Pi-Anion)	−7.7	4.44 × 10^5^

The Diosmetin-2LMO complex is stabilized mainly through hydrogen bonding and hydrophobic interactions (Figure 1D). The amino acid residues of 2LMO, namely I:GLY38:HN (2.77 Å), D:ALA30:O (2.48 Å), and I:GLY38:O (2.05 Å) formed three hydrogen bonds with Diosmetin (Table 2). In addition, D:ILE31 (5.32 Å) and J:VAL39 (3.52 Å, 4.42 Å, and 5.23 Å) interacted with Diosmetin through one and three hydrophobic interactions, respectively. The 2LMO-Diosmetin complex was further stabilized by van der Waals' interactions with B:ASN27, B:GLY29, C:GLY29, C:ILE31, D:LYS28, D:GLY29, E:GLY29, E:ALA30, E:ILE31, H:GLY38, I;GLY37, I:VAL39, I:VAL40, and J:GLY38. The binding energy of 2LMO-Diosmetin complex formation was estimated to be −8.5 kcal mol^−1^ while the dissociation constant was 1.72 × 10^6^ M^−1^ (Table 4).

The 2LMO–Luteolin complex was stabilized by hydrogen bonding and hydrophobic interactions. Luteolin formed four hydrogen bonds with I:GLY38:HN (2.84 Å), E:GLY29:O (2.57 Å), E:GLY29:O (2.38 Å), and C:GLY29:CA (3.36 Å). Also, Luteolin interacted with C:ILE31 (5.49 Å), J:VAL39 (3.57 Å), J:VAL39 (4.43 Å), D:ILE31 (5.22 Å), and J:VAL39 (5.36 Å) through four hydrophobic interactions (Figure 1E). Some residues, such as B:ASN27, B:GLY29, D:LYS28, D:GLY29, D:ALA30, E:ALA30, E:ILE31, H:GLY38, I:VAL39, J:GLY37, I:VAL40, and J:GLY38, are further stabilized 2LMO–Luteolin complex by van der Waals' interactions. The binding free energy and the corresponding dissociation constant of 2LMO-Luteolin were−8.5 kcal mol^−1^ and 1.72 × 10^6^ M^−1^ (Table 4).

#### Interaction of 6TI5 With Phytochemicals

**Figure 2:**
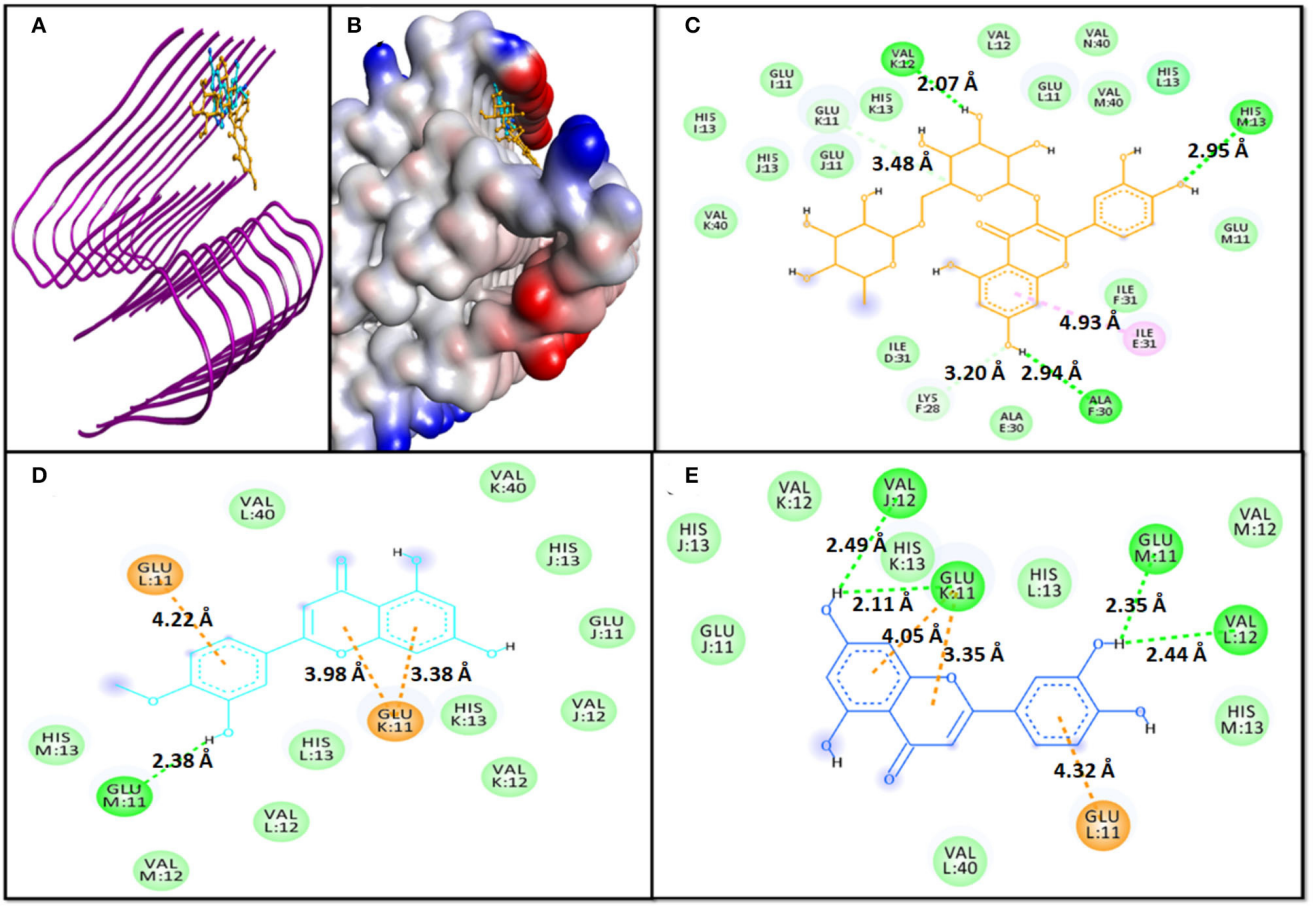
Molecular docking of 6TI5 with phytochemicals. **(A)** 2D representation of the binding of phytochemical to 6TI5, **(B)** 3D representation of the binding of phytochemical to 6TI5, **(C)** Interaction between 6TI5 and Rutin, **(D)** Interaction between 6TI5 and Diosmetin, and **(E)** Interaction between 6TI5 and Luteolin.

In the case of molecular docking with 6TI5, Rutin, Diosmetin, and Luteolin were found to occupy the cavity created due to the formation of fibril i.e., 6TI5 model (Figures 2A,B). It has been found that Rutin interacted with 6TI5 through three conventional hydrogen bonds, two carbon hydrogen bonds, and one hydrophobic interaction with E:ILE:31 (4.93 Å). The conventional hydrogen bonds were formed by M:HIS31:ND1 (2.95 Å), F:ALA30:O (2.94 Å), and K:VAL12:O (2.07 Å), while carbon hydrogen bonds were formed by F:LYS28:CE (3.20 Å), and K:GLU11:OE2 (3.48 Å) (Figure 2C). The 6TI5-Rutin interaction was further stabilized by D:ILE:31, E:ALA30, F:ILE31, I:GLU11, I:HIS13, J:GLU11, J:HIS13, K:HIS13, K:VAL40, L:GLU11, L:VAL12, L:HIS13, M:GLU11, M:VAL40, and N:VAL40, through van der Waals' interaction. Moreover, the binding free energy of Rutin-6TI5 interaction was estimated as −8.5 kcal mol^−1^, and the corresponding binding affinity was 4.44 × 10^5^ M^−1^ (Table 4).

The 6TI5-Diosmetin interaction was favored by one conventional hydrogen bond and three electrostatic interactions (Pi-Anion). The hydrogen bond was formed by M:GLU11:O (2.38 Å), while the electrostatic interactions were formed by K:GLU11:OE2 (3.98 Å, 3.38 Å, and 4.22 Å) (Figure 2D). Further, the Diosmetin-6TI5 complex was stabilized by van der Waals' interactions with residues J:GLU11, J:VAL12, J:HIS13, K:VAL12, K:HIS13, K:VAL40, L:VAL12, L:HIS13, L:VAL40, M:VAL12, and M:HIS13. The binding free energy and the corresponding binding affinity of Diosmetin-6TI5 interaction were−7.7 kcal mol^−1^ and 4.44 × 10^5^ M^−1^ (Table 4).

The 6TI5-Luteolin interaction was favored by four hydrogen bonds and three electrostatic interactions (Pi-Anion). The hydrogen bond was formed by L:VAL12:O (2.44 Å), M:GLU11:O (2.35 Å), and J:VAL12:O (2.49 Å). Likewise, three electrostatic interactions were formed by K:GLU11:OE2 (4.05 Å, 3.35 Å, and 4.32 Å) (Figure 2E). Further, the Luteolin-6TI5 complex was stabilized by van der Waals' interactions with residues J:GLU11, J:HIS13, K:VAL12, K:HIS13, L:HIS13, L:VAL40, M:VAL12, and M:HIS13. The binding free energy and the corresponding binding affinity of Luteolin-6TI5 interaction were −7.7 kcal mol^−1^ and 4.44 × 10^5^ M^−1^ (Table 4).

### Molecular Dynamics Simulation Analysis

#### Root Mean Square Deviation and Root Mean Square Fluctuation Analysis

**Figure 3:**
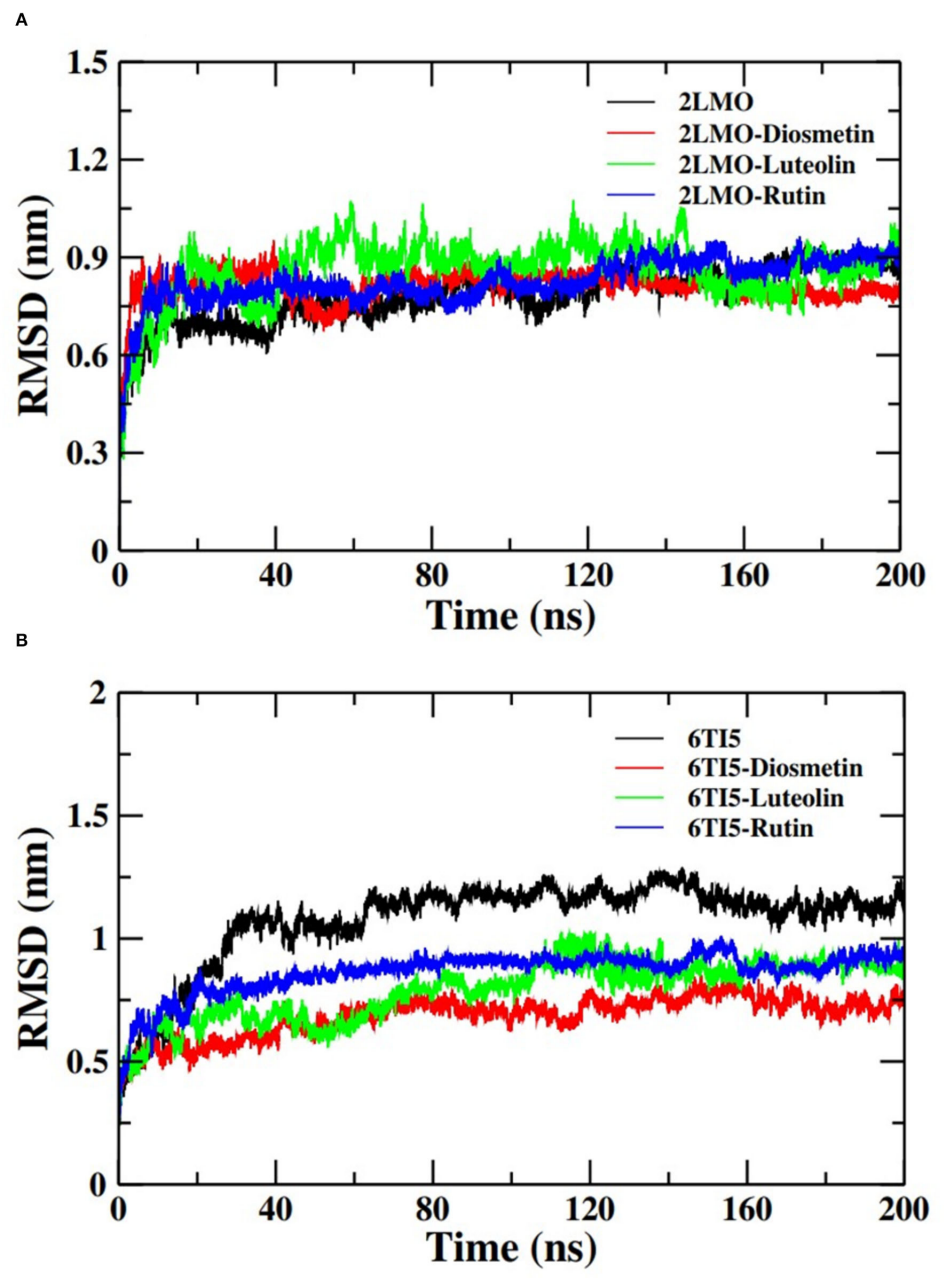
Root mean square deviation (RMSD) of the backbone atoms of **(A)** 2LMO and its complexes with Diosmetin, Luteolin and Rutin and **(B)** 6TI5 and its complexes with Diosmetin, Luteolin and Rutin over 200 ns of MD simulation.

The docked complexes of the 2LMO protein with Diosmetin, Luteolin, and Rutin were simulated in an aqueous environment to study their dynamics and stability. RMSD is a measure of deviation in the initial frame of protein or protein-ligand complex that occurred during the course of MD simulation. The RMSD plot of 2LMO and 6TI5 protein and their complexes with Diosmetin, Luteolin, and Rutin are shown in Figure 3. The RMSD of the backbone atoms of each system was calculated for preliminary analysis of the MD simulation data. The RMSD was calculated with respect to their respective initial conformations. The RMSD of 2LMO depicts some variations initially but was found to be stable after 50 ns of simulation. The 2LMO–Diosmetin complex was found to be stable after 60 ns of simulation time. Similarly, the 2LMO–Luteolin and 2LMO–Rutin complexes attained stability in their structural deviation after 40 and 20 ns of simulation time, respectively. The average RMSD of 2LMO, 2LMO-Diosmetin, 2LMO–Luteolin, and 2LMO-Rutin were found to be at 0.78, 0.81, 0.85, and 0.82 nm, respectively. Further, the RMSD of the backbone atoms of 6TI5 was found to be stable after 35 ns of MD simulation. Similarly, the three complexes of 6TI5 with diosmetion, Luteolin, and Rutin were found to be stable after 20 ns of simulation time. The average RMSD of 6TI5, 6TI5-Diosmetin, 6TI5-Luteolin, and 6TI5-Rutin were calculated to be 1.07, 0.68, 0.78, and 0.86 nm, respectively. However, RMSD plots revealed relatively greater values that attain equilibration after reaching a particular magnitude of deviation. This reason for such higher RMSD values can be further explained by the residue RMSF plot (Supplementary Figures 1, 2). The RMSF plot clearly indicates higher fluctuations in the residues ranging from 20 to 30 amino acids, as well as the C and N terminal residues of 2LMO and 6TI5. These regions define the loop of the protein and exhibit relatively greater RMSF values in some of the chains. This change in RMSF values (relatively higher or lower) is the plausible reason for structural deviations in the respective complexes, resulting in high RMSD values. Several studies have reported a higher value of RMSD of the backbone atoms of Abeta-1-40 protein (Minicozzi et al., 2014; Turner et al., 2019). The native structure of Aβ_1−40_ shows higher RMSD (1.4–1.6 nm), which is its characteristic property Aβ_1−40_ (Minicozzi et al., 2014). In a similar study, RMSD of Aβ_1−40_ was shown to fall in the range of around 1 nm (Turner et al., 2019). Aligning to these previously reported findings, the RMSD values in our study for the Aβ_1−40_ show a comparable range which is stable over the entire course of MD simulation. Moreover, the high RMSD values are also due to fluctuations (RMSF) contributed by the loop regions of the protein as explained earlier. In brief, the data from both the plots clearly indicate that the structures subjected to molecular dynamic simulations attain stability in the aqueous medium after a certain period of simulation time and there isn't any major structural deviation over the course of 200 ns of MD simulation.

#### Radius of Gyration Analysis

**Figure 4:**
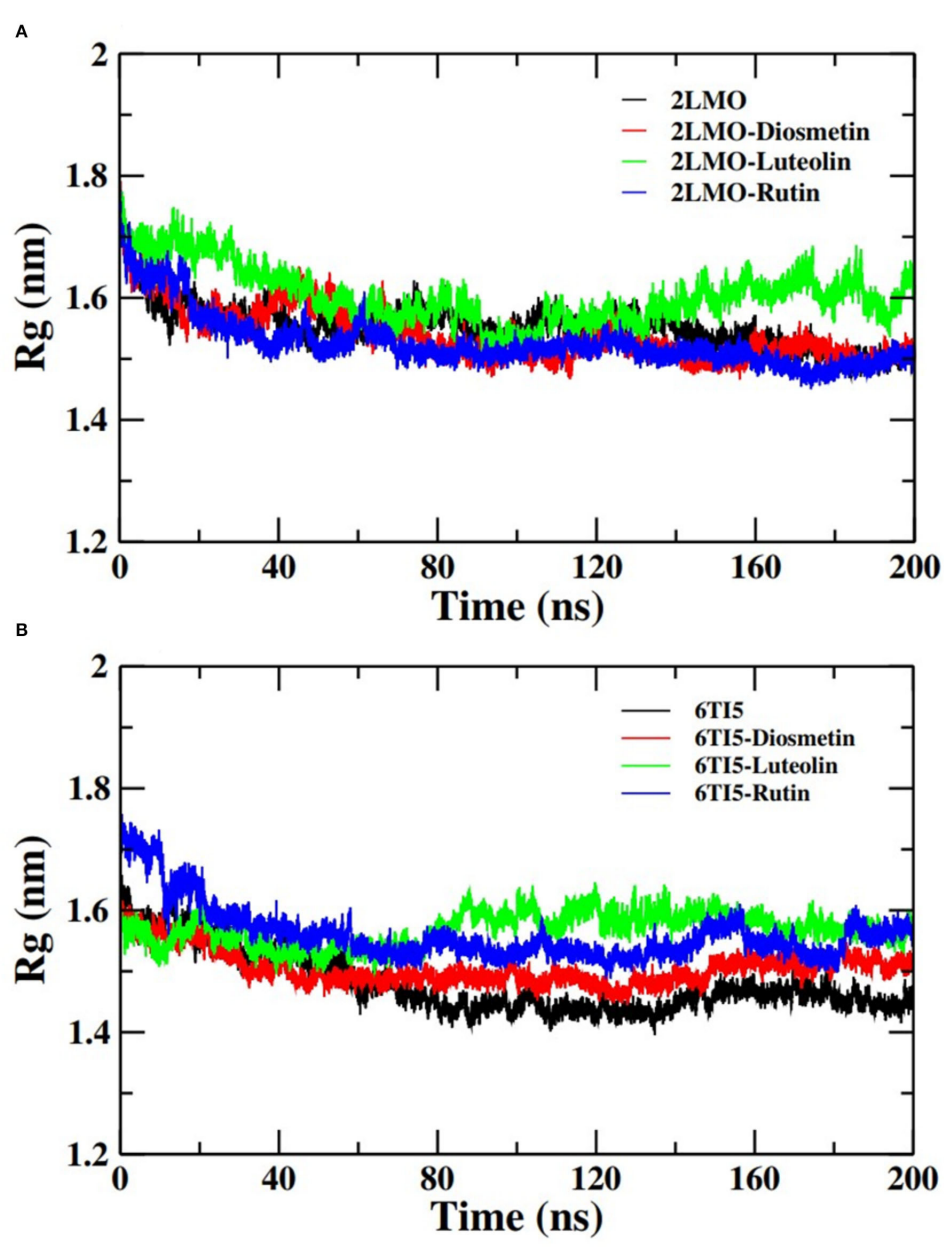
Radius of gyration (Rg) of backbone atoms of **(A)** 2LMO and its complexes with Diosmetin, Luteolin and Rutin and **(B)** 6TI5 and its complexes with Diosmetin, Luteolin and Rutin over the course of 200 ns of simulation time.

Moving forward, the analysis proceeded with calculating the Rg of the C_α_ atoms of the 2LMO and 6TI5 protein and its complex with Diosmetin, Luteolin, and Rutin (Figure 4). Rg indicates the stability of the complexes as a function of the collective mass-weighted root mean square distance of atoms during the molecular dynamic simulation from the center of mass. It is a measure of the overall compactness and 3-D structure of a protein in different conditions and is generally used to access the conformational and folding behavior of proteins (Hashmi et al., 2021). The average Rg of 2LMO, 2LMO-Diosmetin, 2LMO-Luteolin, and 2LMO-Rutin was found to be 1.55, 1.54, 1.60, and 1.52 nm, respectively. Similarly, the average Rg for the 6TI5, 6TI5-Diosmetin, 6TI5-Luteolin, and 6TI5-Rutin was found to be 1.48, 1.50, 1.57, and 1.56 nm, respectively. It should be pointed out that the change in Rg values was not very significant, indicating the formation of stable protein-ligand complexes, conferring the stability of the complexed systems over the course of MD simulations.

#### Solvent Accessible Surface Area Analysis

**Figure 5:**
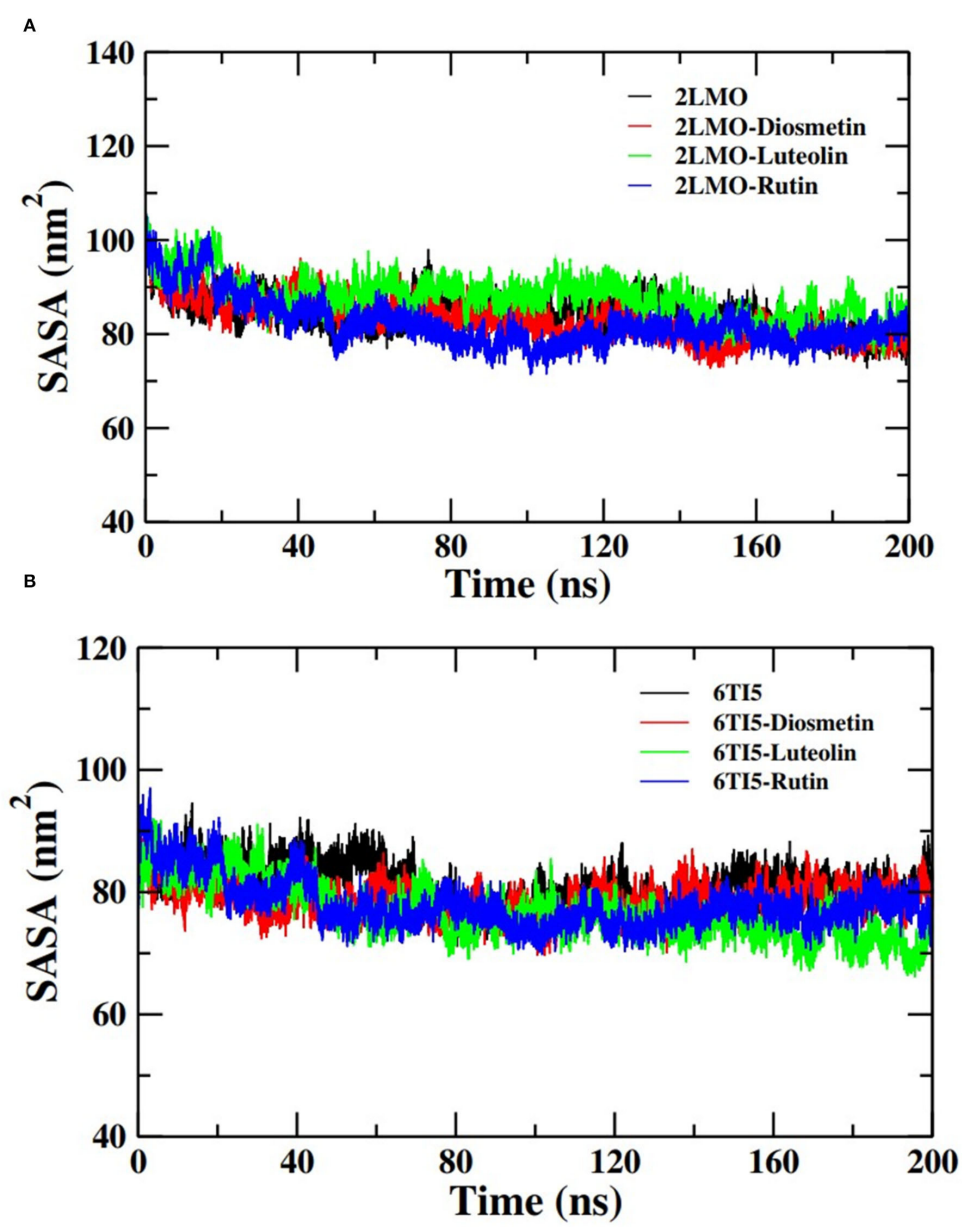
Solvent accessible surface area (SASA) of **(A)** 2LMO and its complexes with Diosmetin, Luteolin and Rutin and **(B)** 6TI5 and its complexes with Diosmetin, Luteolin and Rutin over the course of 200 ns of simulation time.

**Figure 6:**
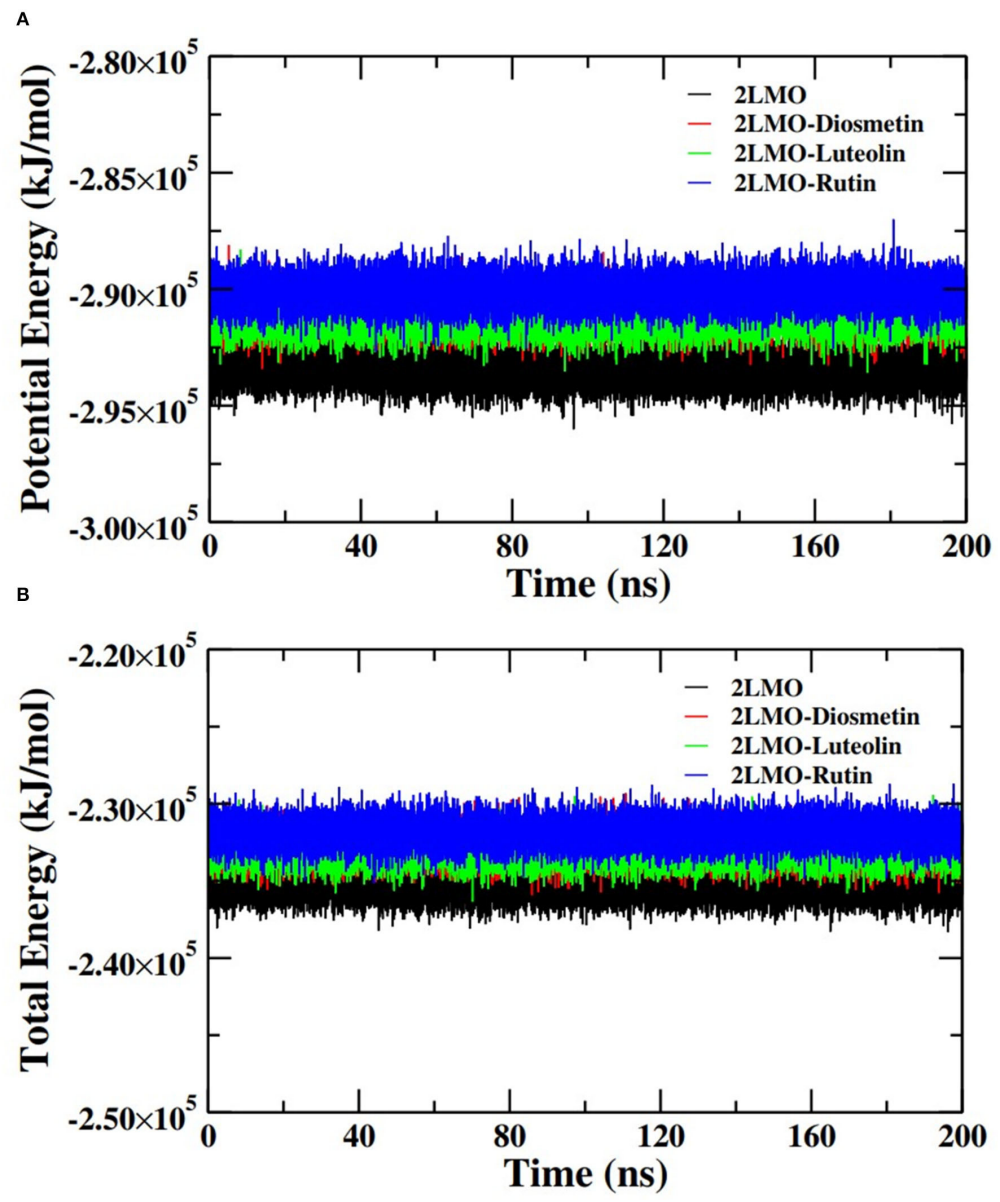
**(A)** Potential energy (PE) and **(B)** total energy (TE) of the systems of 2LMO and its complexes with Diosmetin, Luteolin and Rutin as a function of time.

**Figure 7:**
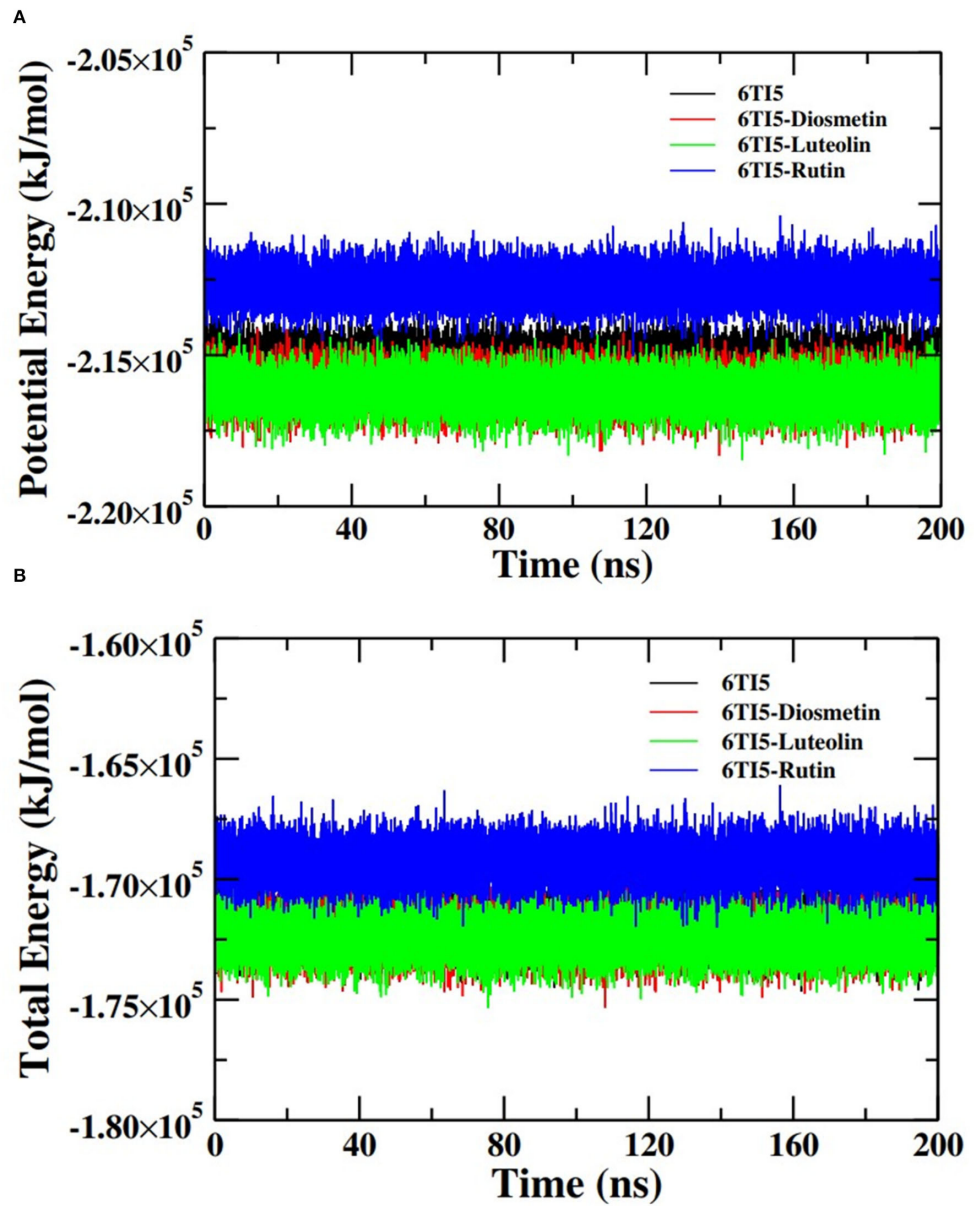
**(A)** Potential energy (PE) and **(B)** total energy (TE) of the systems of 6TI5 and its complexes with Diosmetin, Luteolin and Rutin as a function of time.

Further analysis of the MD simulation data was performed by calculating SASA and the energies of all systems. SASA is a parameter to analyze the nature of structural compactness of proteins and their complexes with the ligand molecules by calculating the area of the protein surface interacting with the continuum solvent (Ahmad et al., 2021). The measurement of SASA is fundamental to understanding the folding-unfolding pathway of a protein in an altered environment or due to the binding of ligand molecules. Here, we have measured SASA of 2LMO and 6TI5 in the presence of Rutin and Diosmetin (Figure 5). The SASA of 2LMO, 2LMO-Diosmetin, 2LMO-Luteolin, and 2LMO-Rutin complexes was found to be constant throughout the simulation. The average SASA of 2LMO, 2LMO-Diosmetin, 2LMO-Luteolin, and 2LMO-Rutin was determined as 84.28, 83.30, 87.73, and 82.30 nm^2^, respectively. Similarly, the SASA of 6TI5 and its complexes with the three ligand molecules were found to be uniform throughout the course of the MD simulation. The average SASA of 6TI5, 6TI5-Diosmetin, 6TI5-Luteolin, and 6TI5-Rutin were found to be 81.05, 78.74, 76.81, and 78.00 nm^2^, respectively. The data shows the stable nature of the proteins (2LMO and 6TI5) with all their complexes in aqueous conditions suggesting that the structure has not compacted or expanded significantly. Moreover, the physicochemical parameters, such as potential and total energies of the system, were also calculated. The total and potential energies of the systems (Figures 6, 7) for both the proteins (2LMO and 6TI5) and their respective complexes with Diosmetin, Luteolin, and Rutin remained constant throughout the simulation, further verifying the stable nature of all systems.

#### Hydrogen Bond Analysis

The interaction of the ligand molecules Diosmetin, Luteolin, and Rutin with 2LMO and 6TI5 proteins was studied by determining the hydrogen bond profiles between the respective complexes (Table 4). The residues, including Gly(A), Gly(B), Val(B), Tyr(C), and His(D), in the 2LMO–Diosmetin complex exhibit 12.86, 9.76, 75.43, 13.05, and 6.54% existence of hydrogen bond formation over the course of 200 ns of MD simulation. Similarly, in case of 2LMO–Luteolin complex, residues Met(A), Gly(A), Gln(B), and Gln(C) contributes with 10.46, 12.44, 8.55, 8.27% existence for H-bond formation. The Val(B) in the 2LMO-Luteolin complex shows more than 40 % H-bond existence. Further, the residues Gly(A), Gln(C), and Tyr(C) in the 2LMO-Rutin complex exhibit 66.87, 32.75, and 61.42% existence of H-bond formation. Val(A) also contributes significantly with >40% H-bond existence in the 2LMO-Rutin complex.

We also studied the hydrogen bond existence map of 6TI5-Diosmetin, 6TI5-Luteolin, and 6TI5-Rutin. Glu22 in the 6TI5-Diosmetin complex shows >15% H-bond existence and Phe19 has 7.44% of H-bond existence. Similarly, in the 6TI5-Luteolin complex, Glu11 and Glu22 show H-bond formation having 5.71 and 7.42% existence. There is significant H-bond formation observed in the 6TI5-Rutin complex. His13 and Gly37 show H-bond formation exhibiting 61.5 and 42.18% existence. In addition, Gln15 also shows >25% of H-bond existence in the 6TI5-Rutin complex. The high percent of hydrogen bond formation between the 2LMO-Rutin and 6TI5-Rutin complexes is due to more polar functional groups in Rutin compared to disometin and Luteolin which order facilitates more possibility of hydrogen bond formation with the protein residues.

#### Evaluation of the Energies Involved in Binding (MM-PBSA Calculations)

The different binding energies involved in the interaction of both the proteins (2LMO and 6TI5) with the respective ligand (Diosmetin, Luteolin, and Rutin) molecules were determined using MM-PBSA calculations. For MM-PBSA calculations, 500 frames were taken out from the last 50 ns MD simulation trajectory of 2LMO and its three complexes at uniform intervals. Similarly, 500 frames from the last 50 ns were extracted for three complexes with 6TI5 protein. The protein-ligand interactions are influenced by the non-covalent forces. These forces include van der Waals forces, hydrophobic interactions, hydrogen bonds, and electrostatic interactions. These forces have either a positive or negative contribution to the overall binding (Siddiqui et al., 2019). The binding energies for the interaction of 2LMO with the respective ligand molecules (Diosmetin, Luteolin, and Rutin) at subsequent intervals of 10 ns are enlisted in Table 5. The binding of all ligands is mostly favored by van der Waals forces and electrostatic interactions. Moreover, there is very less contribution of solvent accessible surface area energy in the interaction of the ligand molecules with the protein (2LMO). On contrary, polar solvation energy impaired the binding of three compounds with the protein. Since polar solvation energy is the energy due to the interaction of the solute with the continuum solvent. Therefore, more the polar functional groups in the ligand molecules (for e.g., Rutin), more will be the polar solvation energy, thereby having a negative contribution to the collective binding energy of the complexes. Among the three complexes of 2LMO, 2LMO-Rutin shows an effective and stronger binding affinity compared to the other two complexes of 2LMO. Similarly, for the complexes of 6TI5, MM-PBSA calculations were performed and the calculated energies at an interval of 10 ns are shown in Table 6. As evident from the data, Rutin has a greater binding for 6TI5 compared to the other two (Diosmetin, Luteolin) molecules. Therefore, Rutin among the three ligand molecules is considered to be an effective drug molecule possessing an efficient binding affinity for the two (2LMO and 6TI5) target proteins.

**Table 5:** Binding free energies (kJ/mol) determined by MM-PBSA calculations of the last 50 ns of trajectories of 2LMO in complex with Rutin, Diosmetin, and Luteolin.

**Binding free energies**	**150–160 ns**	**160–170 ns**	**170–180 ns**	**180–190 ns**	**190–200 ns**
2LMO-Rutin					
ΔE_vdW_	−244.22 ± 20.32	−243.00 ± 21.05	−253.71 ± 22.52	−263.23 ± 23.41	−261.63 ± 21.14
ΔE_ele_	−179.66 ± 36.67	−213.85 ± 31.05	−235.88 ± 37.97	−213.93 ± 52.09	−201.20 ± 31.87
ΔE_PSE_	330.08 ± 28.84	357.82 ± 31.47	377.45 ± 30.82	360.24 ± 40.64	346.89 ± 24.97
ΔE_SASA_	−25.21 ± 1.28	−25.26 ± 1.06	−26.49 ± 1.06	−26.30 ± 1.02	−26.60 ± 1.08
ΔE_BE_	−119.01± 18.42	−124.30 ± 19.53	−138.64 ± 21.85	−143.23 ± 16.63	−142.54 ± 19.12
2LMO-Diosmetin					
ΔE_vdW_	−172.29 ± 13.71	−161.60 ± 11.07	−158.18 ± 11.53	−162.90 ± 15.21	−167.30 ± 12.34
ΔE_ele_	−83.97 ± 38.55	−31.85 ± 12.01	−26.40 ± 14.70	−22.82 ± 13.35	−17.11 ± 11.64
ΔE_PSE_	180.94 ± 38.84	119.51 ± 8.19	117.27 ± 11.02	115.35 ± 11.20	119.54 ± 10.77
ΔE_SASA_	−16.40 ± 0.80	−16.01 ± 0.81	−16.09 ± 0.76	−16.26 ± 0.78	−16.44 ± 0.79
ΔE_BE_	−91.72 ± 15.02	−89.95 ± 12.47	−83.39 ± 11.01	−86.62 ± 13.44	−81.32 ± 14.68
2LMO-Luteolin					
ΔE_vdW_	−97.80 ± 17.03	−90.46 ± 13.11	−83.20 ± 15.60	−62.91 ± 26.20	−75.76 ± 26.63
ΔE_ele_	−102.83 ± 19.60	−92.10 ± 15.55	−101.58 ± 10.68	−53.50 ± 44.37	−53.87 ± 44.70
ΔE_PSE_	153.07 ± 24.38	120.43 ± 17.30	135.88 ± 10.67	85.55 ± 51.90	96.36 ± 42.40
ΔE_SASA_	−13.45 ± 1.17	−12.01 ± 0.68	−12.14 ± 0.92	−9.24 ± 3.12	−9.83 ± 2.14
ΔE_BE_	−61.02 ± 16.93	−74.15 ± 13.51	−61.04 ± 12.32	−40.09 ± 22.59	−43.12 ± 17.26

*ΔE_vdW_, van der Waal energy; ΔE_ele_, Electrostatic energy; ΔE_PSE_, Polar solvation energy; ΔE_SASA_, Solvent accessible surface area energy; ΔE_BE_, Binding energy*.

**Table 6:** Binding free energies (kJ/mol) determined by MM-PBSA calculations of the last 50 ns of trajectories of 6TI5 in complex Diosmetin, Luteolin, and Rutin.

**Binding free energies**	**150–160 ns**	**160–170 ns**	**170–180 ns**	**180–190 ns**	**190–200 ns**
6TI5-Rutin					
ΔE_vdW_	−174.24 ± 1.71	−184.51 ± 1.51	−178.18 ± 1.52	−175.50 ± 1.50	−184.40 ± 1.52
ΔE_ele_	−80.49 ± 2.66	−89.77 ± 2.39	−100.94 ± 2.68	−81.68 ± 1.41	−71.00 ± 1.23
ΔE_PSE_	191.27 ± 3.38	222.09 ± 3.70	227.91 ± 3.33	200.65 ± 1.40	185.82 ± 1.87
ΔE_SASA_	−18.00 ± 0.12	−18.85 ± 0.15	−18.73 ± 0.12	−18.14 ± 0.13	−18.54 ± 0.13
ΔE_BE_	−81.16 ± 2.71	−70.68 ± 2.77	−69.82 ± 1.72	−74.68 ± 1.51	−88.14 ± 1.40
6TI5-Diosmetin					
ΔE_vdW_	−79.50 ± 1.04	−78.52 ± 1.18	−79.31 ± 1.23	−78.50 ± 1.17	−71.57 ± 1.50
ΔE_ele_	−43.93 ± 1.62	−58.86 ± 2.11	−48.42 ± 2.26	−59.50 ± 2.47	−57.52 ± 3.12
ΔE_PSE_	66.76 ± 1.74	81.94 ± 1.61	72.01 ± 2.19	90.62 ± 3.26	89.59 ± 3.28
ΔE_SASA_	−9.20 ± 0.10	−9.54 ± 0.07	−9.57 ± 0.08	−9.82 ± 0.07	−9.39 ± 0.09
ΔE_BE_	−65.80 ± 1.62	−64.97 ± 1.47	−65.29 ± 2.02	−57.20 ± 2.19	−48.83 ± 2.83
6TI5-Luteolin					
ΔE_vdW_	−83.69 ± 0.89	−77.34 ± 0.91	−81.71 ± 1.12	−71.58 ± 1.11	−67.91 ± 1.43
ΔE_ele_	−20.45 ± 0.84	−24.58 ± 0.94	−24.34 ± 0.96	−55.64 ± 4.47	−107.00 ± 5.08
ΔE_PSE_	87.77 ± 1.38	77.07 ± 1.24	82.24 ± 1.52	110.67 ± 4.44	176.81 ± 5.40
ΔE_SASA_	−9.50 ± 0.08	−9.26 ± 0.07	−9.67 ± 0.11	−9.80 ± 0.11	−11.34 ± 0.12
ΔE_BE_	−25.82 ± 1.08	−34.13 ± 1.10	−33.54 ± 1.01	−26.20 ± 1.22	−9.41 ± 1.50

*ΔE_vdW_, van der Waal energy; ΔE_ele_, Electrostatic energy; ΔE_PSE_, Polar solvation energy; ΔE_SASA_, Solvent accessible surface area energy; ΔE_BE_, Binding energy*.

## Conclusion

Based on binding energies and non-bonded interactions, as well as molecular dynamics simulation, Rutin and Luteolin emerged as better lead molecules than Diosmetin. However, high MW (610.5), lowest absorption rate (16.04%), and more than one violation of Lipinski's rule make Rutin a less likely candidate as an anti-amyloidogenic agent. Moreover, among non-violators of Lipinski's rule, Diosmetin exhibited a greater absorption rate than Luteolin as well as the highest positive scores for drug likeness, while Luteolin exhibited moderate drug-likeness. Thus, we can conclude that Diosmetin and Luteolin may serve as a scaffold for the design of better inhibitors with higher affinities toward the target proteins. Our study may open a new vista for the analysis of the neuroprotective potential of these candidate drugs through *in vitro* and *in vivo* techniques.

## Data Availability Statement

The raw data supporting the conclusions of this article will be made available by the authors, without undue reservation.

## Author Contributions

Conceptualization: QZ. Methodology: QZ, MR, SS, and MH. Software: MR, SS, and MH. Validation: QZ and MR. Data analysis: MR, SS, AB, MZA, and AJ. Writing—original draft preparation: QZ, MR, MH, AB, AJ, and MZA. Writing—review and editing: QZ, MR, SS, AJ, SB, and MO. Writing—revision: QZ, HA, SB, II, SA, MA, YA, and WA. Visualization: QZ, MZA, and MFA. Supervision: QZ and MO. Project administration: QZ, AB, SB, and MFA. Funding acquisition: QZ, II, SA, MA, YA, and WA. All authors have read and agreed to the published version of the manuscript.

## Funding

This research was funded by Deputyship for Research and Innovation, Ministry of Education in Saudi Arabia, Grant Number IFP-2020-40.

## Conflict of Interest

The authors declare that the research was conducted in the absence of any commercial or financial relationships that could be construed as a potential conflict of interest.

## Publisher's Note

All claims expressed in this article are solely those of the authors and do not necessarily represent those of their affiliated organizations, or those of the publisher, the editors and the reviewers. Any product that may be evaluated in this article, or claim that may be made by its manufacturer, is not guaranteed or endorsed by the publisher.
