# Supplementary material for: Effect of Date Palm (Phoenix dactylifera) Phytochemicals on Aβ1−40 Amyloid Formation: An in-silico Analysis
Source: Front Neurosci. 2022 Jul 25;16:915122. doi: 10.3389/fnins.2022.915122 (PMC9359633; doi:10.3389/fnins.2022.915122)
Supplement: Supplementary file 1 [file Data_Sheet_1.docx]

**
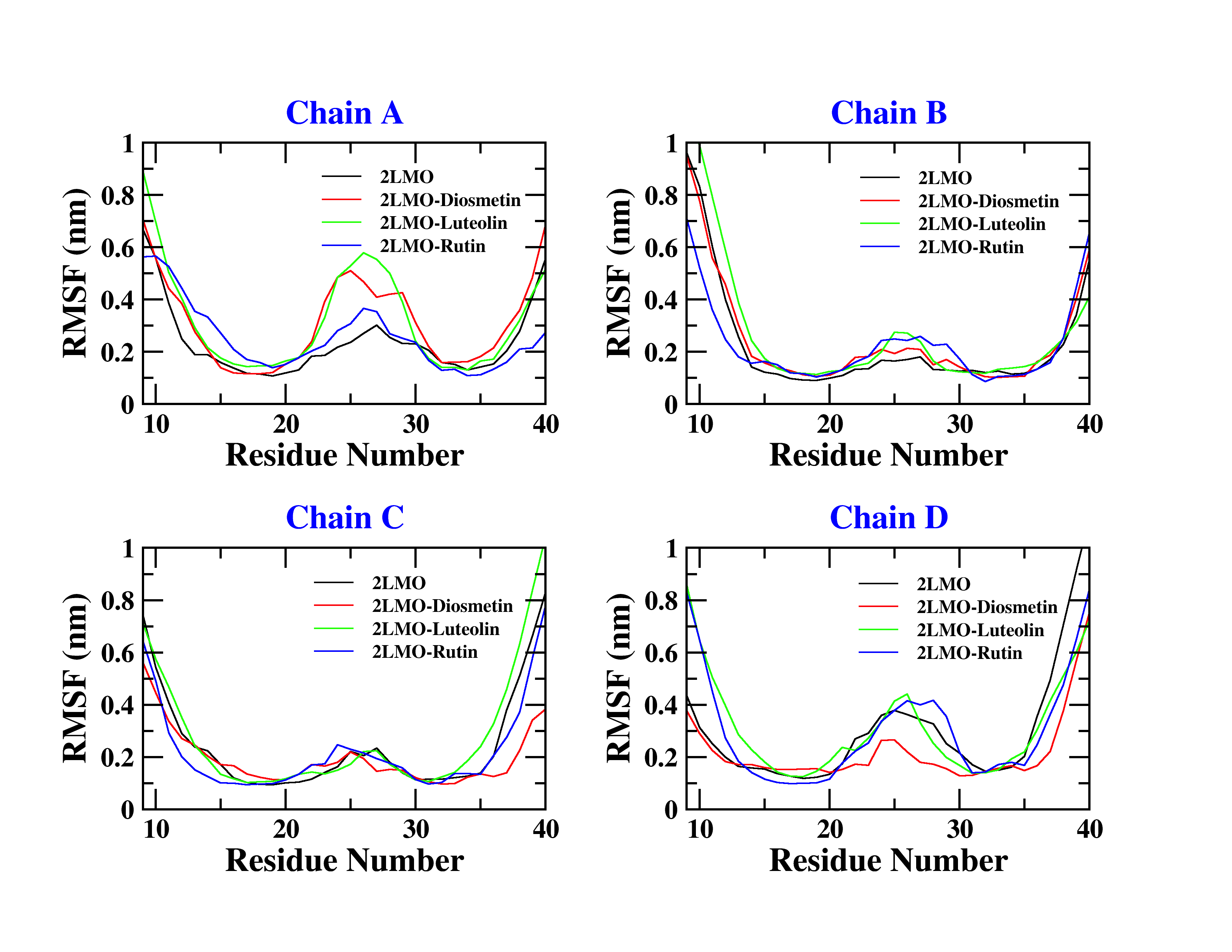
**

**Supplementary figure S1:** Root mean square fluctuation (RMSF) of backbone atoms of 4 chains (tetramer) of 2LMO and their respective complexes with Diosmetin, Luteolin and Rutin.

**
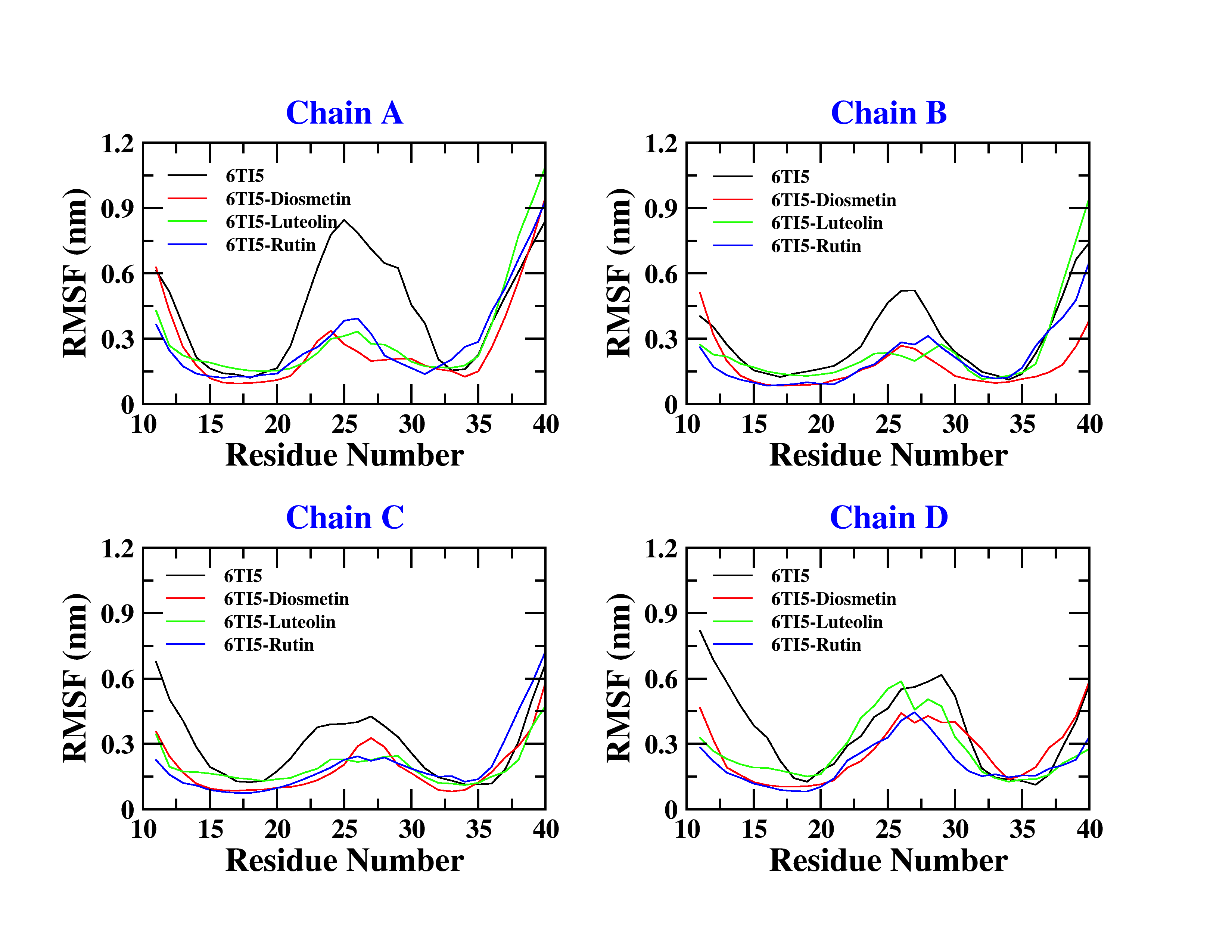
**

**Supplementary figure S2:** Root mean square fluctuation (RMSF) of backbone atoms of 4 chains (tetramer) of 2LMO and their respective complexes with Diosmetin, Luteolin and Rutin.
